# Supplementary material for: Development of Hydroxyapatite Coatings for Orthopaedic Implants from Colloidal Solutions: Part 1—Effect of Solution Concentration and Deposition Kinetics
Source: Nanomaterials (Basel). 2023 Sep 17;13(18):2577. doi: 10.3390/nano13182577 (PMC10535049; doi:10.3390/nano13182577)
Supplement: Supplementary file 1 [file nanomaterials-13-02577-s001.zip › nanomaterials-2585086-supplementary.pdf]

Supplemental Material

Development of Hydroxyapatite Coatings for Orthopaedic Implants from Colloidal Solutions: Part 1—Effect of Solution Concentration and Deposition Kinetics

Bríd Murphy [1,2,*], Mick A. Morris [1,2,*] and Jhonattan Baez [1,2]

[1] Advanced Materials & Bioengineering Research Centre (AMBER), Trinity College Dublin, Dublin 2, D02 CP49 Dublin, Ireland
[2] School of Chemistry, Trinity College Dublin, Dublin 2, D02 PN40 Dublin, Ireland
* Correspondence: murphb52@tcd.ie (B.M.); morrism2@tcd.ie (M.A.M.)

1.0 Molarity and Solubility of Chemicals Used.

Table S1: List of Reagents used and their exact CAS number, the molarity (and molality tolerance) to which they were included in the concentrates, and their water solubility at 20°C.

Reagent	Molarity (mM)	Molarity tolerance	Water Solubility at 20°C
KH_2PO_4 CAS# 7778-77-0	40	0.015	222 g/L
TRIS = $(HOCH_2)_3CNH_2$ CAS# 77-86-1	157.16	2	678 g/L
NaCl CAS# 7647-14-5	4683	0.02	317 g/L
$Ca(NO_3)_2$*$4H_20$ CAS# 13477-34-4	36.364	0.015	1,293 g/L

2.0 Brief explanation behind allowing HA samples to Dry in ambient conditions.

We ran studies whereby we deposited HA films onto a functionalized Quartz Crystal microbalance, the change in dissipation of this crystal represents a phase change. We collected dissipation data through the deposition runs (30 min/1800 s) and up to 35 minutes afterwards (4000 s) and it became clear that the HA film

dehydrated 15mins post process run (2700s on the below graph). On this graph there is a sharp peak after 15 min which represent dehydration of the layer.

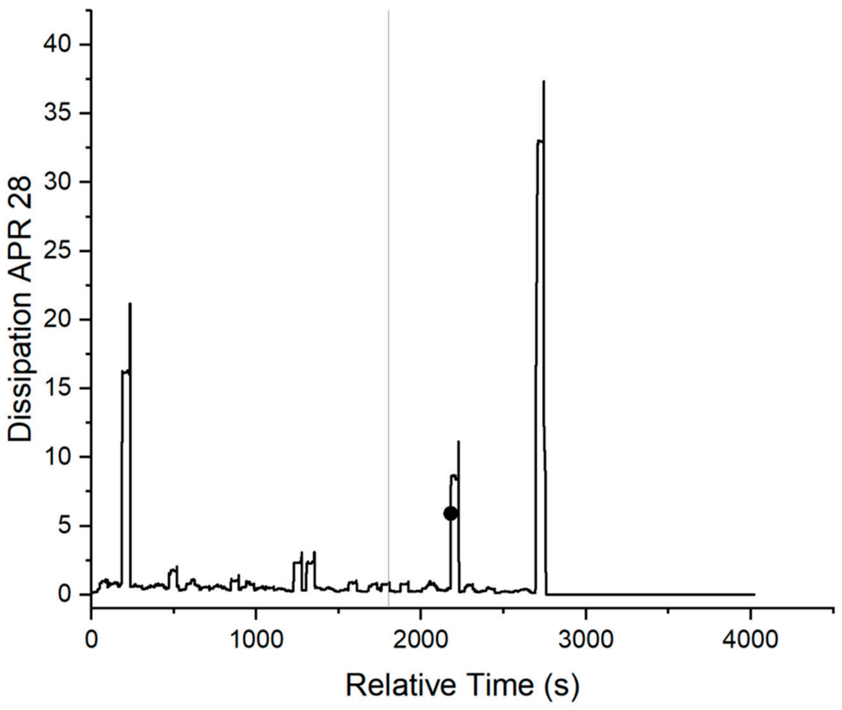

Figure S1: Dissipation versus relative time of a quartz crystal microbalance which has undergone HA deposition, the data shows the sample gathering HA film growth through this colloidal solution process (up to 1800s) and afterwards during drying.

Further to this we carried our studies whereby we deposited HA films onto substrates without letting them dry at all, only rinsing and refilling in between 30 min process runs. Using solution 3, we had the following weights for 6 samples. When we analysed this data reflects a 40% decrease in film growth versus what it could be it parts were allowed to dry in ambient conditions for 15 min.

Table S2: Weight recorded for titanium coupons that were subject to HA deposition without allowing them to dry in between process runs.

Coupon	Before (g)	After 7 runs (g)	weight added (g)
1	7.17	7.1239	-0.0461
2	7.0901	7.0976	0.0075
3	6.9931	6.9999	0.0068
4	6.9649	6.9715	0.0066
5	7.0269	7.0329	0.006
6	6.971	6.9766	0.0056

3.0 Supplemental SEM Images

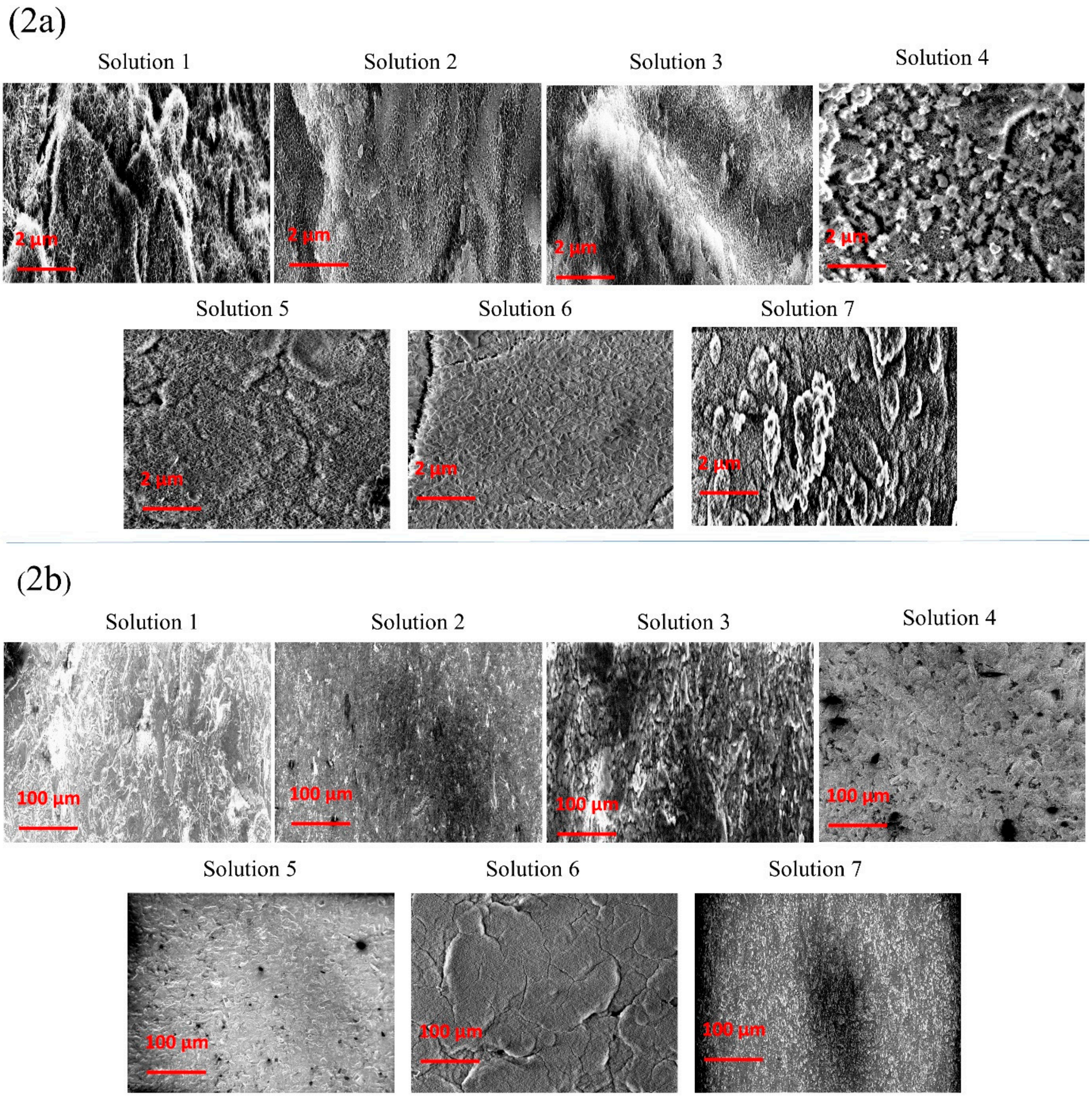

Figure S2: Scanning Electron Microscope (SEM) images of hydroxyapatite film on titanium alloy coupons after 2 hydroxyapatite solution deposition runs using Solutions 1-7. Images

are recorded using a Zeiss Ultra Plus system with the accelerating voltage of 5 kV, at a working distance between 3 to 10 mm and an in-lens detector or secondary electron detector. 2a: SEM images with a scale bar of 2µm to show nature of the porous film. 2b: SEM images with a scale bar of 100µm to show overall sample coverage.

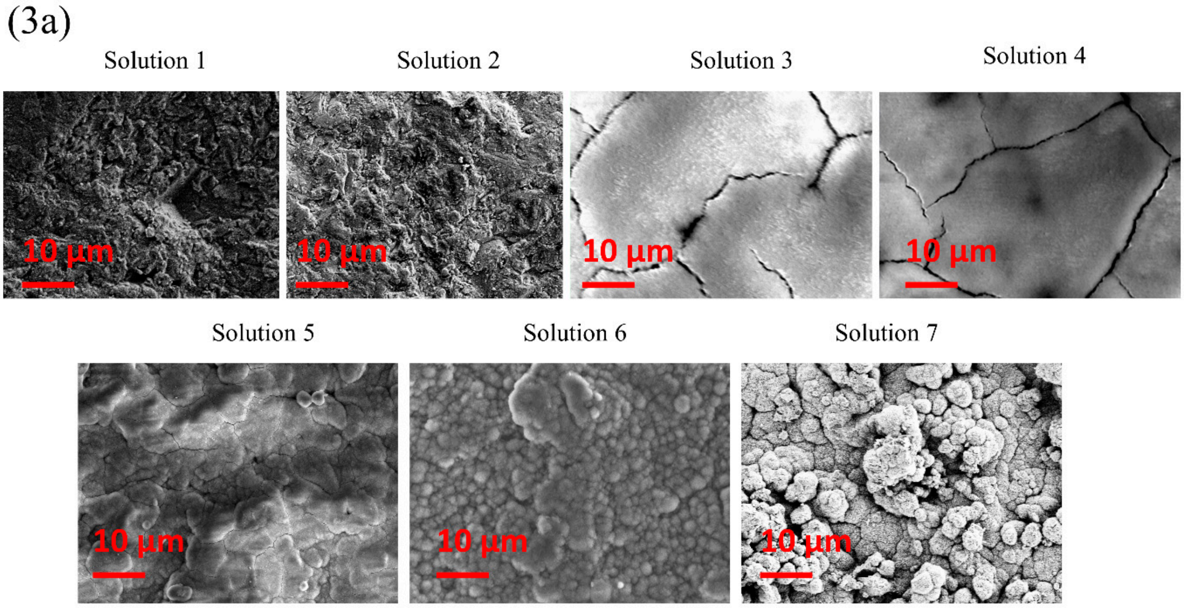

Figure S3: Scanning Electron Microscope (SEM) images of hydroxyapatite film on titanium alloy coupons after 4 hydroxyapatite solution deposition runs using Solutions 1-7. Images are recorded using a Zeiss Ultra Plus system with the accelerating voltage of 5 kV, at a working distance between 3 to 10 mm and an in-lens detector or secondary electron detector, all images have a 10µm scale bar.

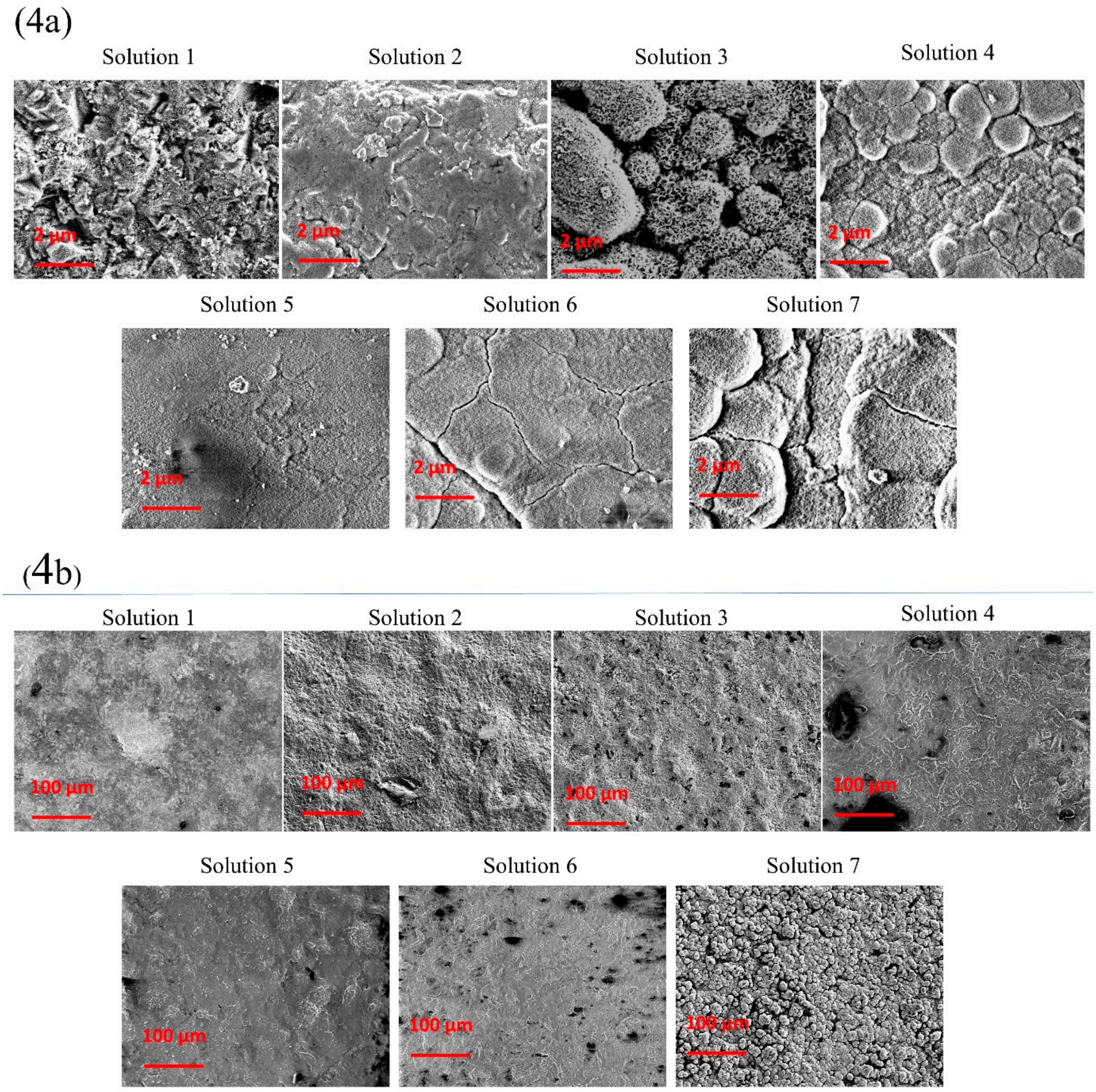

Figure S4: Scanning Electron Microscope (SEM) images of hydroxyapatite film on titanium alloy coupons after 7 hydroxyapatite solution deposition runs using Solutions 1-7. Images are recorded using a Zeiss Ultra Plus system with the accelerating voltage of 5 kV, at a working distance between 3 to 10 mm and an in-lens detector or secondary electron detector. 4a: SEM images with a scale bar of 2μm to show nature of the porous film. 4b: SEM images with a scale bar of 100μm to show overall sample coverage

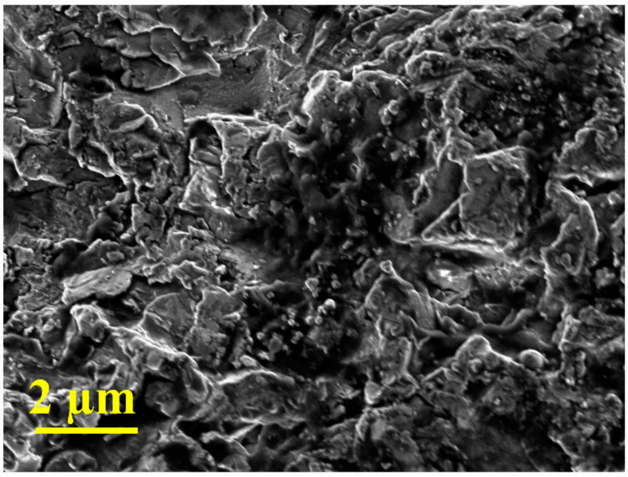

Figure S5: Scanning Electron Microscope (SEM) images of titanium alloy coupons after basic activation. Image recorded using a Zeiss Ultra Plus system with the accelerating voltage of 5 kV, at a working distance of 7.8mm and an in-lens detector.
